# Supplementary material for: ‘The wife should do as her husband advises’: Understanding factors influencing contraceptive use decision making among married Pakistani couples—Qualitative study
Source: PLoS One. 2023 Feb 16;18(2):e0277173. doi: 10.1371/journal.pone.0277173 (PMC9934449; doi:10.1371/journal.pone.0277173)
Supplement: Attachment — Submitted filename: Response to Reviewers.docx [file pone.0277173.s001.docx]

Editor

PLOS One

Submission date (revised manuscript): 11 May 2022

Subject: Revised Manuscript

Dear Editor

I am writing to re-submit our research manuscript entitled, “Decision about the number of children should be made by both the husband and wife; but the wife should do as her husband advises’: Understanding factors influencing contraceptive use decision making among married Pakistani couples” for consideration as a research article for the journal PLOS One. The study explored the perceptions and attitudes of married couples regarding use of modern contraceptives for family planning and child spacing, using a qualitative methodology. The distinct methodology of couples interviews used for this study provided rich data in terms of data on patterns of communications between spouses, on a culturally sensitive issue of family planning. The examination of interactions and dynamics between the couples showed that couples had differing desires about family size and child spacing, while a lack of adequate communication among them led to limited or delayed use of modern contraceptives.

The point by pint response to reviewers’ comments is in the table below.

The authors confirm that this manuscript has not been previously published and is not currently under consideration by any other journal. Additionally, all of the authors have approved the contents of this paper and agree to the Journal’s submission policies. Each author of this manuscript has substantially contributed to conducting the underlying research and drafting this manuscript. Furthermore, to the best of our knowledge, the authors have no conflict of interest, financial or otherwise.

Sincerely,

Mariyam Sarfraz

Response to Comments

| Comment | Action |
| --- | --- |
| Reviewer 1 |  |
| However, what’s missing is an exploration of the tensions between husbands and wives. Further exploration of these areas where there is a lack of alignment how that translates to practices, method choice, and unmet need would add depth to the article. | Thank you for highlighting this important aspect. The discussion section has been amended to explain these findings (Page 24 - 25). |
| Men’s refusal to participate in study was very high and can lend insights into gender dynamics, and the perception/gender allocation of family planning | Thank you for your comment on this part of the study. The discussion section has been amended to explain these findings (Page 23). |
| Large discordance between husband wife with respect to previous contraceptive use, specifically with respect to male methods, what does that also teach us about how masculinity is constructed, what insights how confident in measures of unmet need given limited disclosure, might be worth locating analysis in this larger landscape | Thank you for your comment.  The cultural constructs of men’s role in reproductive health matters and lack of family planning programs for men is included in the discussion section, with relevant references.  (Page 21) |
| Interviews are conducted but husband and wife comments are presented together, please clarify in text | Explanation added in Results section (Page 4). |
| May be worth mentioning the structural limitations that constrain LARC use in introduction/discussion | The limitations to use of LARCs in Pakistan added in the Introduction section (page 2). |
| Reviewer 2 |  |
| - first sentence of the last para on page 15 is missing a word as its not making sense. | Corrected |
| - first sentence on page 26 .....as well has reproductive ...., this should be "as well as reproductive". | Corrected |
| I also think that the title is not defining the results very clearly as its not only husbands who influence contraceptive use decision making, but also other family members and religious beliefs. Looking at the results and discussion, it seems the title may highlight the issue of routinely missing men in contraceptive awareness raising and service provision that leaves them too low in knowledge and understanding to be able to support or even guide their wives. | Thank you for highlighting the gap; agreed that there are several influencers who affect a couple’s decision about family planning. We have discussed these findings considering the available past research and a recent paper of the authors (page 16, references, 31 to 34) in the discussion section. We discussed the title among the research team, and we feel that the title reflects the objective of the study and highlights the disconnect and discord among married couples and their decision making regarding contraceptive use. |
| While describing the sampling and inclusion criteria, it is mentioned that only couples not using LARCs were invited for the qualitative study although they were still using short-acting methods. However, the rationale for this selection is not clarified and if similar criteria has been used by other researchers to identify issues around contraceptive use decision making. | This research paper is based on findings of a sub-study conducted as part of an explanatory mixed methods research conducted with married couples living in rural areas of Islamabad, Pakistan. The sample for this research paper was purposively identified from 800 respondents included in the community-based survey. The reference to the research work has been included in the methods section (reference 11). |
| In addition, it is not mentioned if these couples had any exposure to contraceptive awareness raising programs to determine if their choice of not using LARCs was despite the counseling services or it was due to a weak link in contraceptive counseling programs missing out on husbands and men in general. | The respondent’s knowledge about contraceptives and sources of information are reflected in the results section 1.1 (Lack of functional sexual and reproductive health knowledge). The results show that women received counselling from community health workers (LHWs) and doctors, while the men’s source of knowledge included mainly digital and print media. This is reported in the results section and appropriate quotes are also included. |
