# Supplementary material for: ‘The wife should do as her husband advises’: Understanding factors influencing contraceptive use decision making among married Pakistani couples—Qualitative study
Source: PLoS One. 2023 Feb 16;18(2):e0277173. doi: 10.1371/journal.pone.0277173 (PMC9934449; doi:10.1371/journal.pone.0277173)
Supplement: Attachment — Submitted filename: Response to Comments - October 2022.docx [file pone.0277173.s002.docx]

Response to Reviewers’ Comments

| Comment | Action |
| --- | --- |
| Reviewer 1 |  |
| Authors did not adequately address comments about lacing in gender through the manuscript | We agree that gender has a key role, but, at the same we have been cautious of not extrapolating findings beyond the data and results. The manuscript has been amended to include a discussion on the role of gender in family planning practice of married couples in Pakistan (pages 23 – 24; 27 – 28; 29).  We would also like to state that the objective of this study was to explore the reasons which limited the decision of married couples around use of LARCs. The data collection tool focused more on understanding these factors and did not include enough probes to understand the effect of gender on couple’s approach to family planning and child spacing. |
| no point by point rebuttal letter was provided so unable to understand why this decision was made. | A detailed response letter was prepared and uploaded on the author portal; for reasons unknown to us this was not shared with the reviewers. The response developed is available at the end of this document. |
| Current discussion about cultural context and gender norms is reductive and tends to essentialize cultural norms without making space for differences/discussing nuance | The discussion has been amended to include the role of gender and cultural norms in Pakistan (pages 23 – 24; 27 – 28; 29). |
| Reviewer 3 |  |
| Please highlight the implications for change based on the results you found (the "so what" question for your findings); I would also include that as part of the abstract - given what you found, what would you suggest changing. | Thank you for highlighting the gap in reporting of study findings. The manuscript has been amended to address this (page 2 to 3 and 29) |
| Please emphasize what's different about your study and what your findings add to the current literature. | Unique findings and study implications added in the manuscript (page 2 to 3 and 29). |
| Could you provide more details regarding the translation process in your methods; were the transcripts translated into English and then analyzed? | Methods section has been updated to include the translation and analysis process (page 5). |

Previous response table

| Comment | Action |
| --- | --- |
| Reviewer 1 |  |
| However, what’s missing is an exploration of the tensions between husbands and wives. Further exploration of these areas where there is a lack of alignment how that translates to practices, method choice, and unmet need would add depth to the article. | Thank you for highlighting this important aspect. The discussion section has been amended to explain these findings (Page 24 - 25). |
| Men’s refusal to participate in study was very high and can lend insights into gender dynamics, and the perception/gender allocation of family planning | Thank you for your comment on this part of the study. The discussion section has been amended to explain these findings (Page 23). |
| Large discordance between husband wife with respect to previous contraceptive use, specifically with respect to male methods, what does that also teach us about how masculinity is constructed, what insights how confident in measures of unmet need given limited disclosure, might be worth locating analysis in this larger landscape | Thank you for your comment.  The cultural constructs of men’s role in reproductive health matters and lack of family planning programs for men is included in the discussion section, with relevant references.  (Page 21) |
| Interviews are conducted but husband and wife comments are presented together, please clarify in text | Explanation added in Results section (Page 4). |
| May be worth mentioning the structural limitations that constrain LARC use in introduction/discussion | The limitations to use of LARCs in Pakistan added in the Introduction section (page 2). |
| Reviewer 2 |  |
| - first sentence of the last para on page 15 is missing a word as its not making sense. | Corrected |
| - first sentence on page 26 .....as well has reproductive ...., this should be "as well as reproductive". | Corrected |
| I also think that the title is not defining the results very clearly as its not only husbands who influence contraceptive use decision making, but also other family members and religious beliefs. Looking at the results and discussion, it seems the title may highlight the issue of routinely missing men in contraceptive awareness raising and service provision that leaves them too low in knowledge and understanding to be able to support or even guide their wives. | Thank you for highlighting the gap; agreed that there are several influencers who affect a couple’s decision about family planning. We have discussed these findings considering the available past research and a recent paper of the authors (page 16, references, 31 to 34) in the discussion section. We discussed the title among the research team, and we feel that the title reflects the objective of the study and highlights the disconnect and discord among married couples and their decision making regarding contraceptive use. |
| While describing the sampling and inclusion criteria, it is mentioned that only couples not using LARCs were invited for the qualitative study although they were still using short-acting methods. However, the rationale for this selection is not clarified and if similar criteria has been used by other researchers to identify issues around contraceptive use decision making. | This research paper is based on findings of a sub-study conducted as part of an explanatory mixed methods research conducted with married couples living in rural areas of Islamabad, Pakistan. The sample for this research paper was purposively identified from 800 respondents included in the community-based survey. The reference to the research work has been included in the methods section (reference 11). |
| In addition, it is not mentioned if these couples had any exposure to contraceptive awareness raising programs to determine if their choice of not using LARCs was despite the counseling services or it was due to a weak link in contraceptive counseling programs missing out on husbands and men in general. | The respondent’s knowledge about contraceptives and sources of information are reflected in the results section 1.1 (Lack of functional sexual and reproductive health knowledge). The results show that women received counselling from community health workers (LHWs) and doctors, while the men’s source of knowledge included mainly digital and print media. This is reported in the results section and appropriate quotes are also included. |
